# ARV-825-induced BRD4 protein degradation as a therapy for thyroid carcinoma

**DOI:** 10.18632/aging.102910

**Published:** 2020-03-12

**Authors:** Ling He, Chen Chen, Guoyu Gao, Kun Xu, Zhaoqun Ma

**Affiliations:** 1 Department of General Surgery, Affiliated Hospital of Nanjing University of Chinese Medicine, Jiangsu Province Hospital of Chinese Medicine, Nanjing, China

**Keywords:** thyroid carcinoma, BRD4, ARV-825, proteolysis targeting chimera, c-Myc

## Abstract

Bromodomain-containing protein 4 (BRD4) is overexpressed in thyroid carcinoma, represents as an important therapeutic target. ARV-825 is a novel cereblon-based PROTAC (Proteolysis Targeting Chsimera) compound. It can induce fast and sustained BRD4 protein degradation. Its potential effect in human thyroid carcinoma cells was studied here. In TPC-1 cells and primary human thyroid carcinoma cells, ARV-825 potently inhibited cell viability, proliferation and migration. Furthermore, ARV-825 induced robust apoptosis activation in the thyroid carcinoma cells. ARV-825 induced BRD4 protein degradation and downregulation of its targets, including c-Myc, Bcl-xL and cyclin D1 in thyroid carcinoma cells. It was significantly more potent in inhibiting thyroid carcinoma cells than the known small molecule BRD4 inhibitors. *In vivo* studies demonstrated that ARV-825 oral administration potently suppressed TPC-1 xenograft tumor growth in severe combined immunodeficient mice. BRD4 protein degradation as well as c-Myc, Bcl-xL and cyclin D1 downregulation were detected in ARV-825-treated TPC-1 tumor tissues. Taken together, ARV-825 induces BRD4 protein degradation and inhibits thyroid carcinoma cell growth *in vitro* and *in vivo*.

## INTRODUCTION

Thyroid cancer causes significant human mortalities globally [1, 2]. The primary thyroid cancer form is papillary thyroid carcinoma [1, 2]. Radiation and/or genetic susceptibility are key contributors for the initiation and progression of human papillary thyroid carcinoma [1, 2]. The prognosis and five-year overall survival for the advanced and/or metastatic thyroid carcinoma are extremely poor [1, 2]. Molecularly-targeted therapies are vital for the better treatment of this malignancy [3, 4].

Bromodomain-containing protein 4 (BRD4) is a primary member of the bromodomain and extraterminal domain (BET) family proteins, that contains two bromodomains (BD) and one extraterminal (ET) domain [5–8]. BDs will recognize and interact with lysines in the acetylated histones [9–12]. ET domain recruits transcriptional regulators, promoting expression of several key oncogenes, including *c-myc*, *Bcl-xL* and *Cyclin D1* [9–12]. BRD4 is overexpressed in multiple types of human cancers, emerges as a promising therapeutic oncotarget [9–12].

We have previously shown that BRD4 expression is elevated in thyroid carcinoma cells [13]. Importantly, AZD5153, a novel and specific BRD4 inhibitor, potently inhibited thyroid carcinoma cell growth *in vitro* and *in vivo* [13]. Wang et al., found that gambogic acid downregulated BRD4 to inhibit proliferation of anaplastic thyroid cancer cells [14]. Li et al., demonstrated that long non-coding RNA (LncRNA) UCA1 induced BRD4 upregulation, therefore promoting papillary thyroid cancer cell proliferation [15]. These results imply that BRD4 inhibition might represent as an important therapeutic advance for the treatment of thyroid carcinoma [13].

The small molecule BRD4 inhibitors will, however, lead to feed-back BRD4 protein elevation in cancer cells, resulting in only modest anti-proliferative activity [16]. Lu et al., have recently developed ARV-825 as a heterobifunctional PROTAC (Proteolysis Targeting Chimera) compound. It recruits BRD4 directly to the E3 ubiquitin ligase cereblon [16], leading to fast, efficient and sustained BRD4 protein degradation [16]. Studies have shown that ARV-825 is far more efficient than the small molecule BRD4 inhibitors in suppressing BRD4 signaling, causing potent and sustained cancer cell inhibition and profound apoptosis induction [16–20]. Its potential efficacy in human thyroid carcinoma cells is tested in the present study.

## RESULTS

### ARV-825 inhibits human thyroid carcinoma cell viability, proliferation and migration

**Figure 1:**
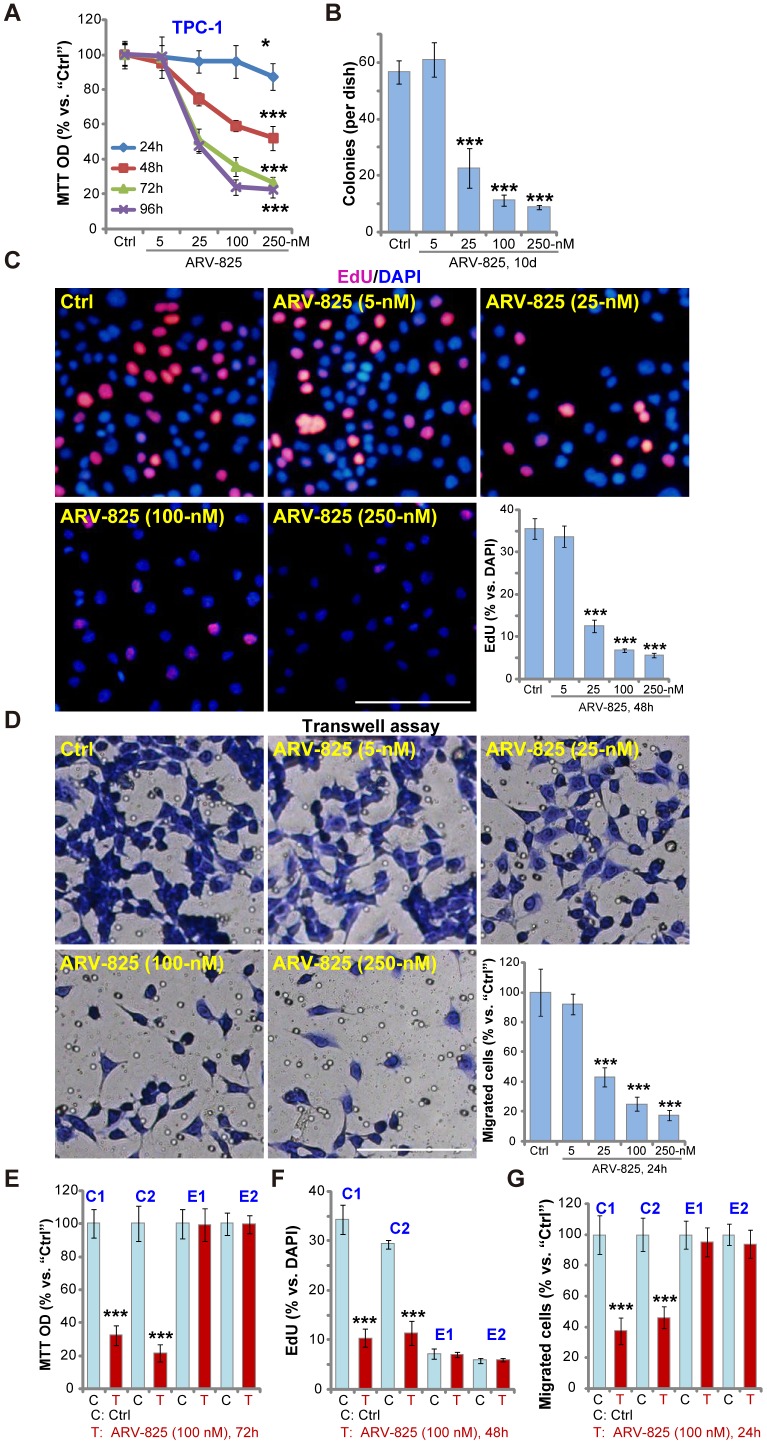
**ARV-825 inhibits human thyroid carcinoma cell viability, proliferation and migration.** TPC-1 cells (**A**–**D**), the primary human thyroid carcinoma cells (“C1”/“C2”, **E**–**G**) or the primary human thyroid epithelial cells (“E1”/“E2”, **E**–**G**) were left untreated (“Ctrl”, same for all Figures) or treated with ARV-825 (5-250 nM). Cells were further cultured in complete medium for indicated time periods, cell viability (MTT OD, **A** and **E**), colony formation (**B**), cell proliferation (EdU incorporation, **C** and **F**) and migration (“Transwell” assays, **D** and **G**) were tested. Data were presented as mean ± standard deviation (SD, n=5) (same for all Figures). *p < 0.05 *vs.* “Ctrl” group. ***p < 0.001 *vs.* “Ctrl” group. The experiments were repeated three times, with similar results obtained. Bar= 100 μm (**C** and **D**).

First, we test the potential effect of ARV-825 on thyroid carcinoma cell functions. The established TPC-1 cells were cultured in complete medium (with FBS), treated with ARV-825 at applied concentrations (5-250 nM). Assaying cell viability, by MTT, demonstrated that ARV-825 inhibited TPC-1 cell viability in a dose-dependent manner (Figure 1A). The cell viability reduction was significant following 25-250 nM of ARV-825 treatments (Figure 1A). At the lowest concentration (5 nM) ARV-825 was ineffective (Figure 1A). The BRD4 PROTAC compound also displayed a time-dependent response in inhibiting TPC-1 cell viability (Figure 1A). The viability started to decline at 24h after ARV-825 (25-250 nM) treatment, being robust at 48-96h (Figure 1A). The colony formation assay results, Figure 1B, demonstrated that ARV-825 dose-dependently decreased the number of viable TPC-1 cell colonies (10 days after first ARV-825 treatment), being significant at 25-250 nM (Figure 1B).

EdU incorporation was analyzed to test cell proliferation. As shown ARV-825, at 25-250 nM, potently decreased EdU incorporation (EdU/DAPI%) in TPC-1 cells (Figure 1C), indicating proliferation inhibition. “Transwell” assay results demonstrated that the number of migrated TPC-1 cells decreased significantly following ARV-825 (25-250 nM, 24h) treatment (Figure 1D). ARV-825 at 5 nM again failed to suppress TPC-1 cell proliferation (Figure 1C) and migration (Figure 1D), showing a dose-dependent response.

The potential effect of ARV-825 in the primary human cells was tested next. As reported early [13], the primary human thyroid carcinoma cells, derived from two papillary thyroid carcinoma patients (“C1/C2”), were treated ARV-825 (100 nM, 24-72h). Results demonstrated that ARV-825 significantly inhibited cell viability (MTT OD, Figure 1E), proliferation (EdU incorporation, Figure 1F) and migration (“Transwell” assay, Figure 1G) in BRD4-overexpressed primary thyroid carcinoma cells [13]. Contrarily, the very same ARV-825 treatment was ineffective in human thyroid epithelial cells (“E1”/”E2”) [13] (Figure 1E–1G), with extremely low BRD4 expression [13]. Collectively, these results demonstrated that ARV-825 potently inhibited thyroid carcinoma cell viability, proliferation and migration.

### ARV-825 induces apoptosis activation in human thyroid carcinoma cells

**Figure 2:**
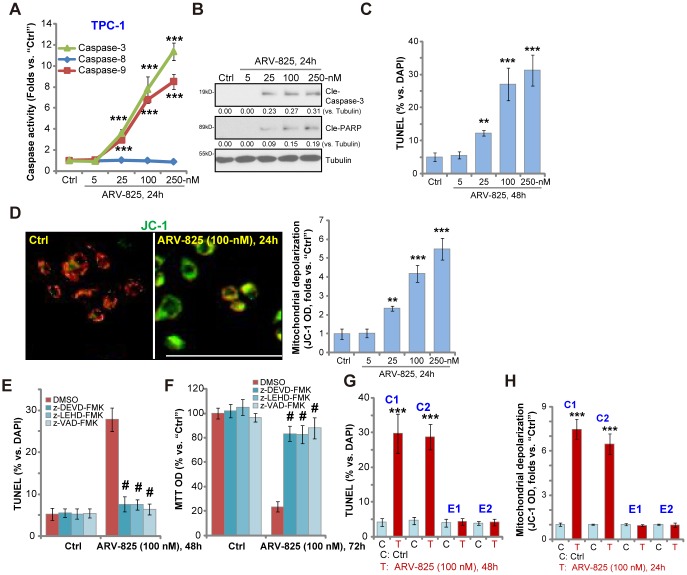
**ARV-825 induces apoptosis activation in human thyroid carcinoma cells.** TPC-1 cells (**A**–**D**), the primary human thyroid carcinoma cell (“C1”/“C2”, **G** and **H**) or the primary human thyroid epithelial cells (“E1”/“E2”, **G** and **H**) were treated with ARV-825 (5-250 nM) and cultured in for indicated time periods, the caspase activity (**A**), expression of apoptosis-associated proteins (**B**) as well as nuclear TUNEL staining (**C** and **G**) and mitochondrial depolarization (JC-1 green fluorescence intensity, D and H) were tested to examine cell apoptosis. TPC-1 cells were pre-treated for 30 min with 50 μM of z-DEVD-fmk, z-LEHD-fmk or z-VAD-fmk, following by ARV-825 (100 nM) treatment for 48-72h, cell apoptosis and cell viability were tested by TUNEL staining (**E**) and MTT assay (**F**), respectively. Expression of the listed proteins was quantified and normalized to loading control (**B**). “DMSO” stands for vehicle control (0.1% DMSO, **E** and **F**). **p < 0.01 *vs.* “Ctrl” group. ***p < 0.001 *vs.* “Ctrl” group. ^#^ *p* < 0.001 *vs.* “DMSO” group (**E** and **F**). The experiments were repeated three times, with similar results obtained. Bar=100 μm (**D**).

To study whether ARV-825 could induce apoptosis activation in thyroid carcinoma cells, the caspase activity assay was performed. As demonstrated, ARV-825, in a dose-dependent manner, increased activities of caspase-3 and caspase-9 in TPC-1 cells (Figure 2A). The caspase-8 activity, the indicator of apoptosis activation by death receptor pathway, was however unchanged (Figure 2A). Furthermore, ARV-825 treatments (25-250 nM, 24h) induced cleavages of caspase-3 and its downstream PARP (poly (ADP-ribose) polymerase) in TPC-1 cells (Figure 2B). The nuclear TUNEL ratio was also significantly increased in TPC-1 cells with ARV-825 treatments (25-250 nM, 48h) (Figure 2C). Mitochondrial depolarization, evidenced by the JC-1 green fluorescence accumulation, was detected as well in TPC-1 cells after ARV-825 (25-250 nM, 24h) stimulation (Figure 2D), further suggesting apoptosis activation. ARV-825 at 5 nM however failed to induce significant apoptosis activation in TPC-1 cells (Figure 2A–2D).

To test the link between ARV-825-induced apoptosis activation and cytotoxicity in thyroid carcinoma cells, the apoptosis inhibitors were utilized, including the caspase-3 specific inhibitor z-DEVD-fmk, the caspase-9 inhibitor z-LEHD-fmk and the pan-caspase inhibitor z-VAD-fmk. As shown, ARV-825 (100 nM)-induced apoptosis activation (nuclear TUNEL ratio increase, Figure 2E) and cell viability (MTT OD) reduction (Figure 2F) were largely attenuated by the caspase inhibitors (Figure 2E and 2F). These results suggest that caspase-apoptosis activation is responsible for ARV-825-induced cytotoxicity in TPC-1 cells. In the human thyroid carcinoma cells, “C1” and “C2”, ARV-825 (100 nM, 24-48h) treatment induced significant apoptosis activation, evidenced by increased nuclear TUNEL staining (Figure 2G) and mitochondrial depolarization (Figure 2H). It was however non-apoptotic in the primary thyroid epithelial cells, “E1” and “E2” (Figure 2G and 2H). Collectively, these results show that ARV-825 induced robust apoptosis activation in thyroid carcinoma cells.

### ARV-825 inhibits BRD4 signaling in human thyroid carcinoma cells

**Figure 3:**
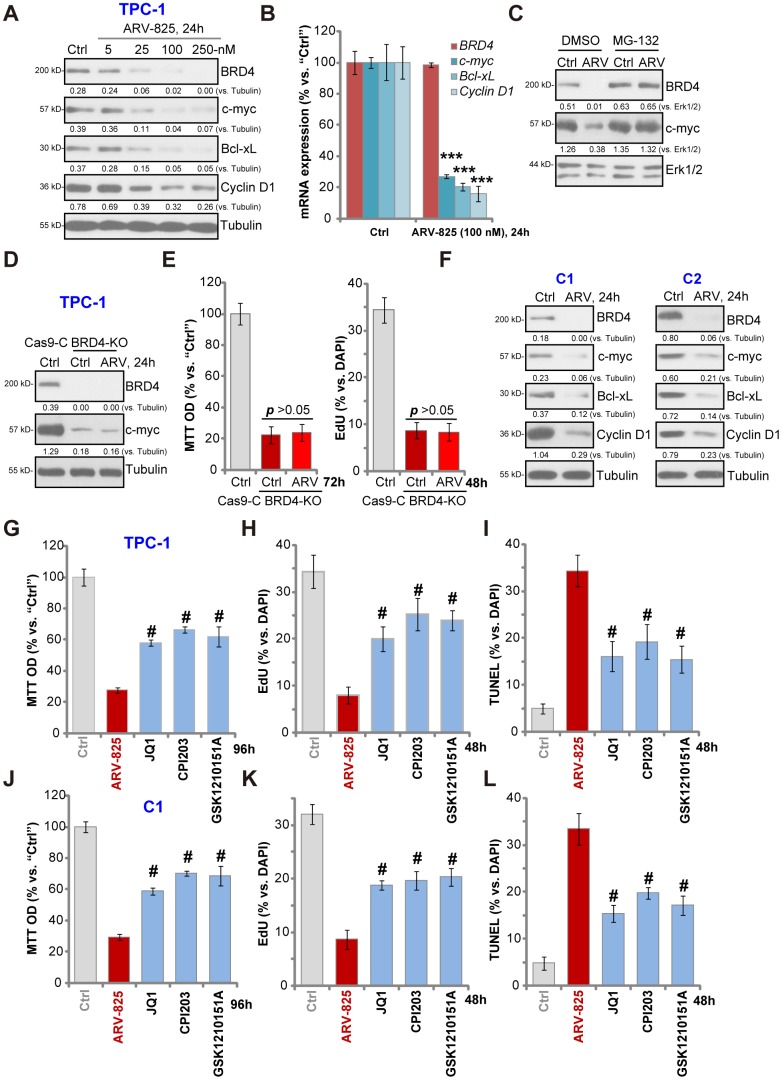
**ARV-825 inhibits BRD4 signaling in human thyroid carcinoma cells.** TPC-1 cells (**A** and **B**) or the primary human thyroid carcinoma cells (“C1”/“C2”) (**F**) were treated with applied concentration of ARV-825 for 24h, tested by Western blotting and qPCR assays of listed genes. TPC-1 cells were pre-treated with MG-132 (25 μM) for 2h, followed by ARV-825 (100 nM) treated for 24h, expression of listed proteins was shown (**C**). The stable TPC-1 cells with CRISPR/Cas9 BRD4-KO construct (“BRD4-KO”) cells were treated with or without ARV-825 (100 nM, for indicated time periods), control TPC-1 cells with empty vector (“Cas9-C”) were left untreated; expression of listed proteins was shown (**D**); Cell viability and proliferation were tested by MTT and EdU staining assays (**E**), respectively. TPC-1 cells or “C1” human thyroid carcinoma cells were treated with ARV-825 (100 nM), JQ1 (500 nM), CPI203 (500 nM) or GSK1210151A (500 nM) for indicated time periods, cell viability (MTT assay, 96h) (**G** and **J**), proliferation (testing nuclear EdU/DAPI ratio, 48h) (**H** and **K**) and apoptosis (nuclear TUNEL ratio, 48h) (**I** and **L**) were tested, and results were quantified. Expression of listed proteins was quantified and normalized to the corresponding loading control (**A**, **C**, **D** and **F**). ^#^ *p* < 0.001 *vs.* “ARV-825” treatment group (**G**–**L**). The experiments were repeated three times, and similar results obtained.

ARV-825’s effect on BRD4 signaling proteins was studied. Western blotting assay results, Figure 3A, demonstrated that ARV-825 dose-dependently decreased BRD4 protein level in TPC-1 cells. BRD4-dependent oncogenic proteins, c-Myc [21], Bcl-xL [22] and cyclin D1 [23, 24], were downregulated as well (Figure 3A). qPCR assay results in TPC-1 cells, Figure 3B, showed that *BRD4 mRNA* was unchanged with ARV-825 treatment (100 nM, 24h). While mRNA levels of *c-Myc*, *Bcl-xL* and *cyclin D1* were significantly reduced (Figure 3B). Importantly, ARV-825-induced BRD4 protein depletion was completely reversed by the protease inhibitor MG-132 (Figure 3C). Furthermore, MG-132 restored c-Myc protein expression in ARV-825-treated TPC-1 cells (Figure 3C). These results implied that ARV-825 induced BRD4 protein degradation, causing downregulation of its target genes (c-Myc, Bcl-xL and cyclin D1) in TPC-1 cells.

Mimicking ARV-825-induced actions, CRISPR-Cas9-induced BRD4 KO (using the previously-described protocol [23], Figure 3D) significantly downregulated c-Myc (Figure 3D), inhibiting TPC-1 cell viability and proliferation (*p* < 0.001 *vs.* control cells) (Figure 3E). Importantly, in the BRD4-KO TPC-1 cells ARV-825 (100 nM) treatment failed to further downregulate c-Myc (Figure 3D) nor to inhibit cell viability/proliferation (Figure 3E). These results implied that ARV-825- induced cytotoxicity in TPC-1 cells should be the result of BRD4 degradation. In the primary papillary carcinoma cells (“C1/C2”), treatment with ARV-825 (100 nM, 24h) similarly induced BRD4 protein degradation as well as downregulation of c-Myc, Bcl-xL and cyclin D1 (Figure 3F). Together, ARV-825 induced BRD4 protein degradation and downregulated its targeted genes in thyroid carcinoma cells.

Unlike the small molecular inhibitors of BRD4, ARV-825 will result in robust and sustained BRD4 protein degradation [18]. We therefore compared its activity with several known BRD4 inhibitors, including JQ1 [25, 26], CPI203 [23] and GSK1210151A [27]. In TPC-1 cells (Figure 3G–3I) and “C1” primary thyroid carcinoma cells (Figure 3J–3L), ARV-825 was significantly more potent than the tested BRD4 inhibitors (at even higher concentrations) in inhibiting cell viability (Figure 3G and 3J) and proliferation (EdU incorporation, Figure 3H and 3K) as well as inducing cell apoptosis (nuclear TUNEL staining, Figure 3I and 3L). Therefore, ARV-825 was highly efficient in inhibiting thyroid carcinoma cell progression *in vitro*.

### ARV-825 oral administration inhibits TPC-1 xenograft tumor growth in SCID mice

**Figure 4:**
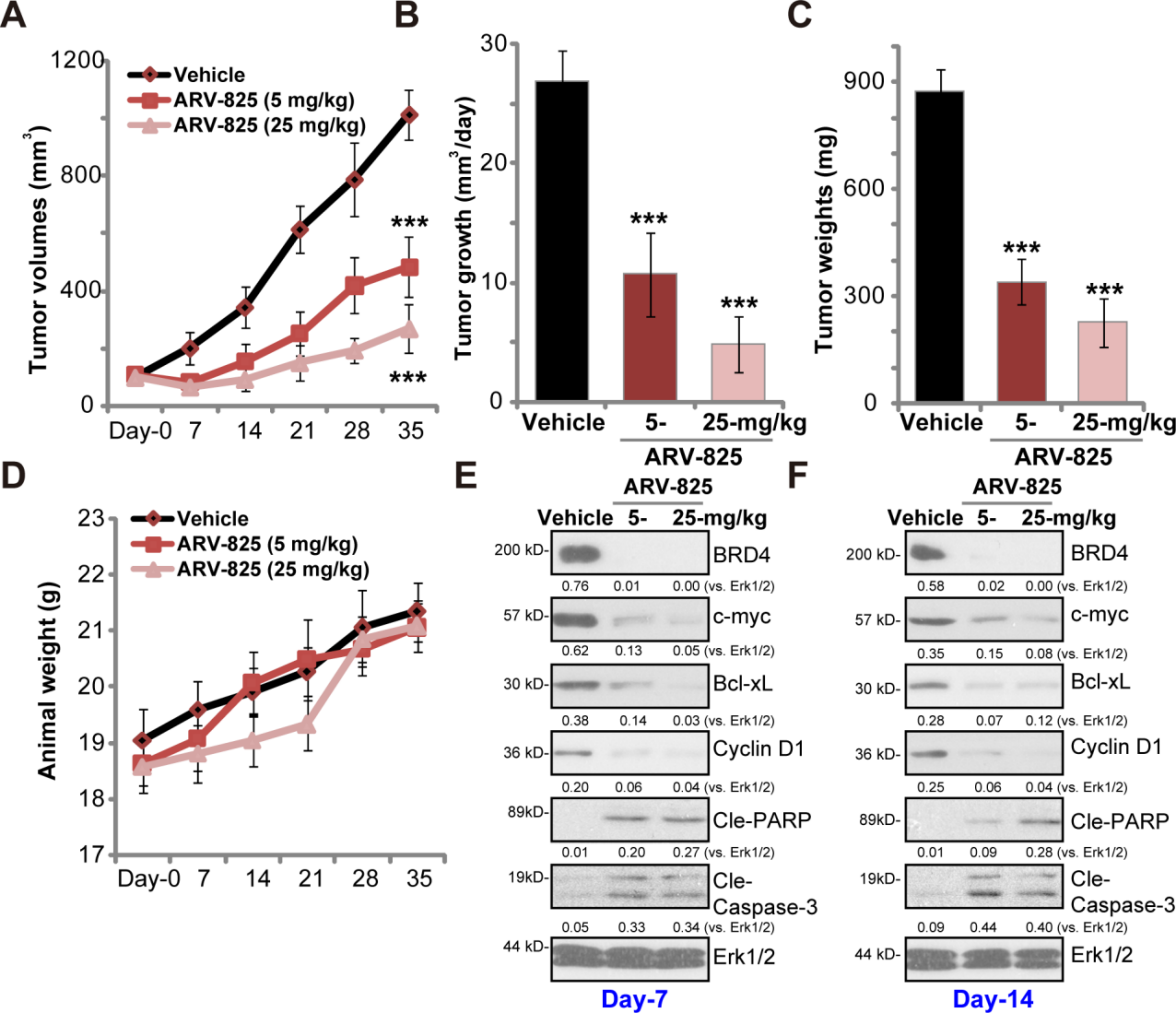
**ARV-825 oral administration inhibits TPC-1 xenograft tumor growth in SCID mice.** SCID mice bearing TPC-1 xenografts (100 mm^3^ at “Day-0”) were treated with ARV-825 (daily gavage, 5 or 25 mg/kg body weight, for 21 consecutive days), the tumor volumes (**A**) and the mice body weights (**D**) were recorded every seven days (recording five rounds). The estimated daily tumor growth (in mm^3^ per day) was calculated (**B**). At “Day-35” tumors of all three groups were isolated and weighted (**C**). At treatment “Day-7” and “Day-14”, one tumor of each group was isolated, with fresh tumor tissue lysates analyzed by Western blotting for listed proteins (**E** and **F**). Expression of listed proteins was quantified and normalized to Erk1/2 (**E** and **F**). Vehicle stands for 10% DMSO, 40% PEG300, 5% Tween-80 plus 45% saline. For each group, n= 10 mice. ***p < 0.001 *vs.* “Vehicle” group.

At last experiments were carried out to test the potential anti-thyroid carcinoma activity of ARV-825 *in vivo*. As described previously [13] TPC-1 cells were *s.c.* injected to the flanks of SCID mice, forming tumor xenografts within 16-18 days (“Day-0”). By measuring tumor volumes, we demonstrated that oral administration of ARV-825 (daily, at 5 or 25 mg/kg body weight) potently inhibited TPC-1 xenograft growth in SCID mice (Figure 4A). ARV-825 administration decreased estimated daily tumor growth, which was calculated by (tumor volume at Day-35—tumor volume at Day-0)/35 (Figure 4B). At Day-35 all xenograft tumors were isolated. Tumors from ARV-825-treated mice weighted significantly lighter than those from vehicle control mice (Figure 4C). ARV-825 at 25 mg/kg dose was more potent in suppressing TPC-1 tumor growth than it at 5 mg/kg dose (Figure 4A–4C). The mice body weights were not significantly different among the three groups (Figure 4D), neither did we notice any signs of apparent toxicities in the experimental mice.

To test BRD4 signaling proteins, at treatment Day-7 and Day-14 one tumor of each group (total six tumors) was isolated, and fresh tumor lysates subjected to Western blotting assays. As demonstrated, BRD4 protein was depleted in ARV-825-treated xenograft tumors (Figure 4E and 4F). BRD4-dependent proteins, c-Myc, Bcl-xL and cyclin D1, were downregulated (Figure 4E and 4F). Cleavages of caspase-3 and PARP were detected in ARV-825-treated tumor tissues (Figure 4E and 4F), indicating apoptosis activation *in vivo*. These results suggest that ARV-825 oral administration in SCID mice inhibited BRD4 signaling and activated apoptosis in TPC-1 xenografts.

## DISCUSSION

BRD4 is the most abundant and important BET family protein in cancer cells [9–11, 28]. It is a key epigenetic regulator binding to acetylated-histones [9–12]. BRD4 is also required for the chromatin structure formation in the daughter cells [9–11]. By recruiting P-TEFb (the positive transcription elongation factor b) and phosphorylating RNA polymerase II, BRD4 is essential for transcription elongation and expression of multiple key oncogenic genes [11], including *Bcl-xL*, *c-Myc*, *cyclin D1*, among others [5, 8–12]. Our previous studies have demonstrated that BRD4 expression is significantly elevated in established and primary human thyroid carcinoma cells [13]. BRD4 inhibition, by the small molecule inhibitor AZD5153, potently inhibited thyroid carcinoma cell growth *in vitro* and *in vivo* [13]. These studies supported that BRD4 is a promising and valuable therapeutic target of thyroid carcinoma.

ARV-825 is a molecule designed by the PROTAC technology, causing sustained BRD4 protein degradation [16]. The results of this study suggest that ARV-825 exerted potent anti-thyroid carcinoma cell activity [16, 18, 19, 22, 29]. In TPC-1 cells and primary human thyroid carcinoma cells ARV-825 induced BRD4 protein degradation, causing downregulation of BRD4-dependent oncogenic proteins, including c-Myc, Bcl-xL and cyclin D1. ARV-825 potently inhibited thyroid carcinoma cell viability, proliferation and migration, and inducing robust cell apoptosis. The BRD4 PROTAC compound was, however, non-cytotoxic nor pro-apoptotic to primary thyroid epithelial cells. *In vivo*, oral administration of ARV-825, at well-tolerated doses, efficiently inhibited TPC-1 tumor xenograft growth in SCID mice. BRD4 protein degradation as well as c-Myc, Bcl-xL and cyclin D1 downregulation were detected in ARV-825-treated TPC-1 tumor tissues. Therefore, BRD4 degradation by its PROTAC compound ARV-825 could be novel therapeutic advance of thyroid carcinoma.

It has been shown that BRD4 inhibitors could have reversible binding and incomplete inhibition of BRD4, which largely compromise the anti-cancer cell activity [16]. ARV-825, unlike BRD4 inhibitors, directly recruits BRD4 to the E3-ubiquitin ligase cereblon, causing robust and sustained BRD4 protein degradation [16]. In the present study, ARV-825-induced anti-thyroid carcinoma cell activity was significantly more potent than the small molecule BRD4 inhibitors, including JQ1, CPI203, and GSK1210151A. Our results suggest that BRD4 protein degradation should be the main reason to explain ARV-825-induced superior anti-thyroid carcinoma cell activity. As BRD4 KO, by the CRSIPR/Cas9 strategy, mimicked ARV-825-induced cytotoxicity in TPC-1 cells. More importantly, ARV-825 was completely ineffective in the BRD4-KO cells.

## CONCLUSION

Taken together, we conclude that ARV-825 induced BRD4 protein degradation and inhibited thyroid. carcinoma cell growth *in vitro* and *in vivo*. It could be an important therapeutic advance for the treatment of thyroid carcinoma.

## MATERIALS AND METHODS

### Chemicals and reagents

ARV-825 was purchased from MCE China (Suzhou, China). MG-132 was obtained from Sigma Chemicals (Beijing, China). The caspase inhibitors, z-DEVD-fmk, z-LEHD-fmk and z-VAD-fmk, were provided by Calbiochem (La Jolla, CA). GSK1210151A, JQ1 and CPI203 were provided by Selleck (Shanghai, China). The antibodies were all purchased from Cell Signaling Tech (Beverly, MA).

### Cell culture

The established TPC-1 human thyroid carcinoma cells were cultured as previously described [13]. TPC-1 cells were routinely checked for possible mycoplasma and microbial contamination. To confirm the the genotype STR profiling, population doubling time, and morphology were verified. The primary human thyroid carcinoma cells (“C1/C2”) as well as the thyroid follicular epithelial cells (“E1/E2”), derived from two primary papillary thyroid carcinoma patients, were reported early [13], and cultured using the described protocol [13]. The protocols of using human cells were in accordance with the Declaration of Helsinki, approved by the Institutional Ethics Review Board of authors institutions.

### Cell viability

As previously described [13], at 5,000 cells per well cells were initially plated into 96-well plates. Following the applied treatment, cell viability was tested by the MTT assay, with its optical density (OD) values measured at the wavelength of 550 nm.

### Colony formation

After the applied ARV-825 treatment THP-1 cells were placed onto 10-cm dishes (20,000 cells/dish) [13]. Cells were maintained in ARV-825-containing complete medium for a total of 10 days (with medium renewed every two days). The number of viable THP-1 cell colonies remained was manually counted.

### Caspase activity

Following the applied ARV-825 treatment, 30 μg of cytosolic protein extracts were mixed with the caspase assay buffer and the caspase (-3/-8/-9) substrate [13]. An Infinite 200-PRO reader was utilized to quantify the release of fluorogenic AFC at 400 nm excitation and 505 nm emission. AFC fluorescence value was always normalized to that of vehicle control.

### TUNEL (terminal deoxynucleotidyl transferase dUTP nick end labeling) staining

Cells were initially seeded into the 12-well plates (5 x 10^4^ cells/cm^2^). With the applied treatment, a TUNEL In Situ Cell Death Detection Kit (Roche) was applied to test cell apoptosis. Briefly, cells were stained with TUNEL and DAPI, visualized under a fluorescent microscope (Leica). In each condition 600 cell nuclei in six random views were counted to calculate TUNEL-positive nuclei ratio (% *vs.* DAPI).

### EdU (5-ethynyl-20-deoxyuridine) staining

Cells were initially seeded into 24-well plates (2 x 10^4^ cells/cm^2^). With the applied treatment, an EdU Apollo-567 assay kit (RiboBio, Guangzhou, China) was utilized to test cell proliferation. Cells were co-stained with EdU and DAPI, visualized under a fluorescent microscope (Leica). In each condition 600 cell nuclei in six random views were counted to calculate EdU/DAPI ratio [30, 31].

### Mitochondrial depolarization

With mitochondrial depolarization JC-1 dye shall aggregate in the mitochondria to form green monomers in the stressed cells [32]. After the applied ARV-825 treatment, cells were incubated with JC-1 (5 μg/mL, Sigma), tested under the fluorescence spectrofluorometer at the test-wavelength of 545 nm (green). The JC-1 fluorescence images, integrating the green (at 550 nm) and red (at 635 nm) channels, were presented as well.

### “Transwell” assay

The thyroid carcinoma cells (10, 000 cells in 250 μL serum-free medium) with the applied ARV-825 treatment were seeded on the upper surface of the “Transwell” chambers (BD Biosciences). FBS-containing complete medium was added to the lower “Transwell” chambers. After incubation for 24h the migrated cells were stained and counted.

### Western blotting

The detailed protocols of Western blotting were described previously [33, 34]. In brief, total cellular lysates (30-40 μg proteins per treatment in each lane) were separated by 10-12% SDS-PAGE gels, transferred to a PVDF blot. After blocking, the blot was incubated with the applied primary and secondary antibodies, with the antibody-antigen binding detected by an ECL kit. For data quantification, the ImageJ software (NIH) was utilized.

### RNA extraction and qPCR

Total RNA was extracted via the TRIzol reagents (Invitrogen Thermo-Fisher). Quantitative real-time reverse transcription-PCR (“qPCR”) assay was carried using the previously-described protocol [23]. *GAPDH* was always examined as the reference gene for data quantification, using the 2^−∆∆*C*t^ method. mRNA primers utilized in this study were provided by Dr. Zhao at Soochow University [23].

### BRD4 knockout (KO)

The lentiCRISPR-BRD4-KO-GFP construct, provided by Dr. Zhao at Soochow University [23], was transfected to TPC-1 cells by Lipofectamine 2000 (Invitrogen Thermo-Fisher). FACS was carried to sort the GFP-positive single cells, with BRD4 KO verified by Western blotting.

### Xenograft assay

The detailed protocols were reported in our previous study [13]. Briefly, from the Experimental Animal Center of Soochow University (Suzhou, China) the severe combined immunodeficient (SCID) mice were obtained. To establish tumor xenografts TPC-1 cells were subcutaneously (*s.c.*) injected to the right flanks [13]. Tumor-bearing SICD mice were randomly assigned into three groups (10 mice per group), treated with ARV-825 or the vehicle control. Tumor volumes and the mice body weights were recorded every seven days. The animal studies were conducted with the standards of ethical treatment approved by the Institutional Animal Care and Use Committee (IACUC) of authors’ institutions.

### Statistical analyses

Data were presented as mean ± standard deviation (SD). Statistics were analyzed by one-way ANOVA followed by the Scheffe' and Tukey Test using SPSS software (21.0, SPSS Co. Chicago, CA). Significance was chosen as *p* < 0.05. To determine significance between two treatment groups, the two-tailed unpaired t tests (Excel 2007) were carried out.
